# Challenges in the design and analysis of a factorial-design cluster randomised trial

**DOI:** 10.1186/1745-6215-16-S2-P149

**Published:** 2015-11-16

**Authors:** Jacqueline Stephen, Robert Lee, Kalliopi Kydonaki, Jean Antonelli, Timothy S Walsh, Christopher J Weir

**Affiliations:** 1 Edinburgh Clinical Trials Unit, University of Edinburgh, Edinburgh, UK; 2 Anaesthetics, Critical Care and Pain Medicine, University of Edinburgh, Edinburgh, UK; 3 Centre for Population Health Sciences, University of Edinburgh, Edinburgh, UK; 4 Edinburgh Health Services Research Unit, Edinburgh, UK

## Introduction

Optimising sedation quality in mechanically ventilated intensive care patients is important because excessive sedation is associated with increased hospital acquired infections, longer intensive care (ICU) and hospital stay, and possibly higher mortality. The Development and Evaluation of Strategies to Improve Sedation Quality in InTensive Care (DESIST) study aims to optimise sedation practice. Here we focus on the study design, statistical analysis plan, performing the analysis and issues that occurred. DESIST randomised eight ICUs in pairs to four different combinations of sedation-related quality improvement interventions. The primary outcome assessed optimum sedation within each 12 hour nursing shift (referred to as a DESIST care period). This resulted in a three-level hierarchical data structure: DESIST care periods within admissions, within ICU.

## Challenges

The detailed analysis plan specified a generalised linear mixed model for the analysis of the primary outcome. Due to the complex hierarchical data structure, we used MLwiN which is specifically designed for the analysis of multilevel models. We will illustrate the use of the STATA *runmlwin* command which allows MLwiN to be run from within STATA.

Modelling ICU as both a fixed and random effect resulted in difficulties in model convergence and estimating the ICU level variance. There was non-normality of residuals and high auto-correlation within MCMC chains.

## Conclusion

The small number of ICUs and lack of evidence of ICU-level clustering caused difficulties in fitting the three-level multilevel model. Fitting a simpler two-level model was feasible, gave similar intervention effect estimates and was justified by the lack of ICU-level clustering.

